# The role of epigenetic methylation/demethylation in the regulation of retinal photoreceptors

**DOI:** 10.3389/fcell.2023.1149132

**Published:** 2023-05-26

**Authors:** Chao-Fan Lu, Ya-Nan Zhou, Jingjing Zhang, Songxue Su, Yupeng Liu, Guang-Hua Peng, Weidong Zang, Jing Cao

**Affiliations:** ^1^ Department of Anatomy, Basic Medical College, Zhengzhou University, Zhengzhou, China; ^2^ Department of Pathophysiology, Basic Medical College, Zhengzhou University, Zhengzhou, China; ^3^ Laboratory of Visual Cell Differentiation and Regulation, Basic Medical College, Zhengzhou University, Zhengzhou, China

**Keywords:** DNA methylation, histone methylation, differentiation, degradation, photoreceptors

## Abstract

Photoreceptors are integral and crucial for the retina, as they convert light into electrical signals. Epigenetics plays a vital role in determining the precise expression of genetic information in space and time during the development and maturation of photoreceptors, cell differentiation, degeneration, death, and various pathological processes. Epigenetic regulation has three main manifestations: histone modification, DNA methylation, and RNA-based mechanisms, where methylation is involved in two regulatory mechanisms-histone methylation and DNA methylation. DNA methylation is the most studied form of epigenetic modification, while histone methylation is a relatively stable regulatory mechanism. Evidence suggests that normal methylation regulation is essential for the growth and development of photoreceptors and the maintenance of their functions, while abnormal methylation can lead to many pathological forms of photoreceptors. However, the role of methylation/demethylation in regulating retinal photoreceptors remains unclear. Therefore, this study aims to review the role of methylation/demethylation in regulating photoreceptors in various physiological and pathological situations and discuss the underlying mechanisms involved. Given the critical role of epigenetic regulation in gene expression and cellular differentiation, investigating the specific molecular mechanisms underlying these processes in photoreceptors may provide valuable insights into the pathogenesis of retinal diseases. Moreover, understanding these mechanisms could lead to the development of novel therapies that target the epigenetic machinery, thereby promoting the maintenance of retinal function throughout an individual’s lifespan.

## 1 Introduction

**FIGURE 1:**
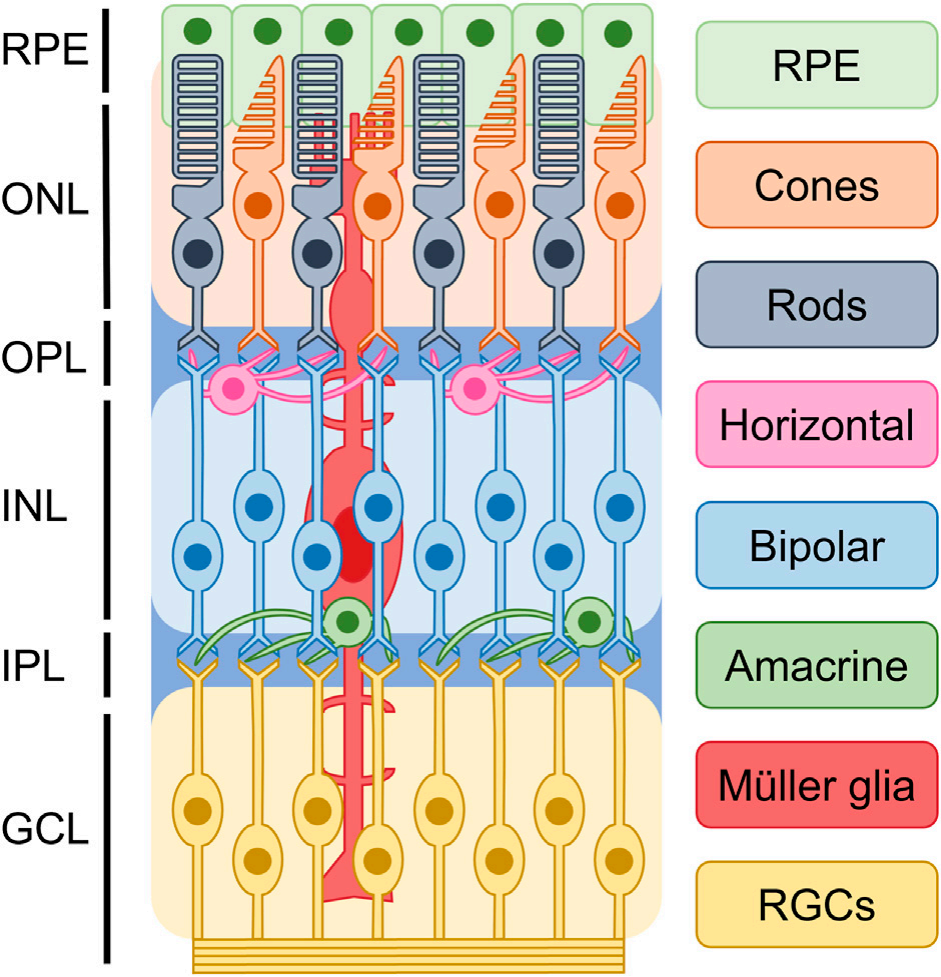
Basic structure of retina. Based on current understanding of the epigenetics of retinal cells, it is possible to classify the retina into distinct cellular types. These include photoreceptors, bipolar cells, retinal ganglion cells (RGCs), horizontal cells, amacrine cells, and glial cells. They are divided into ganglion cell layer (GCL), inner plexiform layer (IPL), inner nuclear layer (INL), outer plexiform layer (OPL), and outer nuclear layer (ONL), retinal pigment epithelium (RPE).

The mammalian retina develops from the forebrain neuroepithelium during the early stages of central nervous system development and serves as the primary site for processing and transmitting visual signals to the brain. As shown in Figure 1, based on cellular typing, the retina can be classified into distinct cell types, including photoreceptors, bipolar cells, retinal ganglion cells (RGCs), horizontal cells, amacrine cells, and glial cells (Hussey et al., 2022). Photoreceptors, which can be classified into cones and rods, are the functionally specialized cells of the retina that play a crucial role in converting light signals to electrical signals. These electrical signals are then processed and transmitted to the brain, where they are translated into visual information (Singh et al., 2017). Rods and cones are two distinct types of photoreceptors that play different functional roles in the retina. Rods are primarily responsible for low-light or scotopic vision, and they do not detect color information. On the other hand, cones are responsible for high visual acuity, bright vision, and color perception or photopic vision (Mo et al., 2016; Hussey et al., 2022). The process of photoreceptor differentiation begins in retinal progenitor cells (RPCs), which undergo proliferation and silencing of precursor genes before expressing photoreceptor-specific genes. These cells then differentiate into photoreceptor precursors, which undergo further maturation to become mature photoreceptors. During this process, axonal growth, synapse formation, and outer segment biogenesis occur (Swaroop et al., 2010). During retinal development, different multipotent retinal progenitors acquire the ability to generate specific neuronal groups in a specific sequence, which is regulated by intrinsic and extrinsic factors. These factors act in combination to guide the differentiation of RPCs towards specific cell fates, ultimately leading to the formation of different retinal cell types (Wallace, 2011).

**FIGURE 2:**
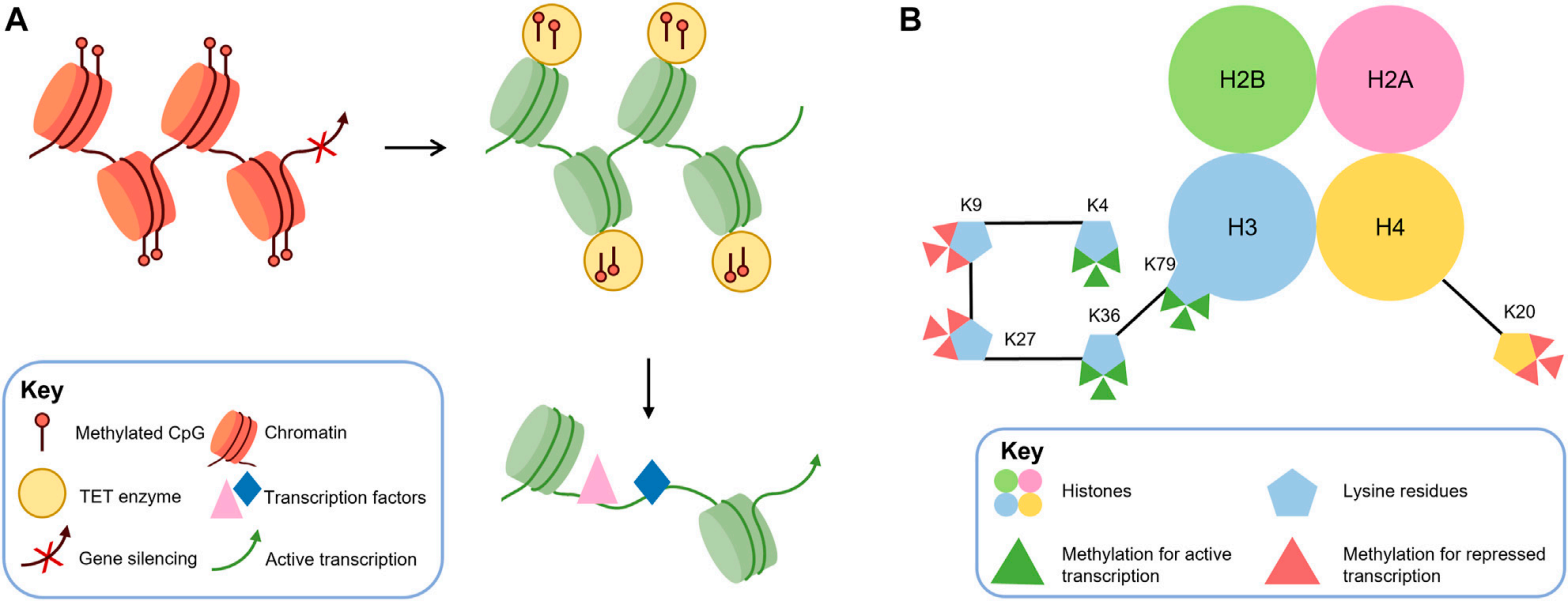
DNA methylation and histone methylation are crucial epigenetic mechanisms that can regulate gene transcription. **(A)** DNA methylation can alter local chromatin structure and reduce accessibility for transcription factors, resulting in decreased transcriptional activity. Conversely, demethylation mediated by TET enzymes can reverse this effect by increasing DNA accessibility and promoting transcription factor binding, leading to enhanced transcriptional activity. **(B)** Histone methylation predominantly occurs at specific lysine (K) residues on histones H3 and H4, resulting in mono-, di-, or trimethylation (me1, me2, and me3). H3K4, H3K36, and H3K79 methylation are generally associated with transcriptional activation. In contrast, H3K9, H3K27, and H4K20 methylation are associated with transcriptional repression.

In recent years, epigenetics has gained attention for its role in various physiological and pathological functions, with DNA methylation being the most extensively studied mechanism. DNA methylation is catalyzed by DNA methyltransferases (DNMTs), which include three main isoforms: DNMT1, DNMT3A, and DNMT3B. DNMT1 maintains DNA methylation patterns during DNA replication, while DNMT3A and DNMT3B generate new DNA methylation patterns in specific differentiated cells and tissues, including the central nervous system (Moore et al., 2013). DNA methylation is a crucial epigenetic mark that plays a critical role in regulating gene expression in the vertebrate genome. In normal conditions, DNA methylation is involved in various processes, including the inactivation of one X chromosome in females, tissue-specific gene expression, and suppression of transposable elements. However, aberrant DNA methylation patterns have been implicated in numerous human disorders, including retinal diseases such as age-related macular degeneration and diabetic retinopathy (Fan et al., 2001; Klein et al., 2011). During mammalian embryonic development, the methylation profile of the entire genome is established by *de novo* DNA methyltransferases (DNMT3A and 3B) and is strictly maintained in subsequent cell divisions by the maintenance methyltransferase DNMT1 (Reizel et al., 2021). During cell differentiation, methylation mediated by TET family demethylases or *de novo* methyltransferases DNMT3A and DNMT3B produces stage-specific and cell-type-specific changes to assist in the differentiation of cells for specific properties and functions (Ziller et al., 2013; Argelaguet et al., 2019). As shown in Figure 2A, DNA methylation can affect changes in local chromatin structure and reduce the accessibility of transcription-related proteins to gene sequences in the region, thereby regulating transcriptional activity (Reizel et al., 2018). DNA demethylation is initiated by specific trans-acting factors with a “pioneer” function and is accomplished by the TET enzyme, which stabilizes the opening of the demethylated region and increases the binding of transcription factors (Zaret, 2020). Epigenetic disorders arising from DNA methylation can have significant impacts on cellular differentiation during development. Such disorders may restrict the differentiation of stem or progenitor cells into specific cell types and also prevent differentiated cells from reverting to their undifferentiated states. In the context of retinal development, aberrant DNA methylation patterns can lead to various pathological conditions, including retinoblastoma and age-related macular degeneration (Corso-Diaz et al., 2018). The process of DNA demethylation is crucial for stem or progenitor cells to differentiate into mature cells. While DNA methylation patterns can restrict cellular differentiation, DNA demethylation can remove such barriers and facilitate the expression of genes necessary for cell maturation. In the retina, proper DNA demethylation is essential for the development of specific retinal cell types, including photoreceptors and bipolar cells (Koh and Rao, 2013; Huang and Rao, 2014). It includes the differentiation of photoreceptors. Therefore, changes in DNA methylation and demethylation can affect the normal differentiation of photoreceptors. DNA demethylation can be achieved in two ways, of which the most studied is the activity of DNA demethylase demethylation (Koh and Rao, 2013; Huang and Rao, 2014). In the active pathway of DNA demethylation, the TET family of DNA methylation erasers is a key regulator of DNA demethylation, which completes the 5mC to 5hmC transition (Tahiliani et al., 2009). In recent years, the TET family has played an increasingly important role in the tissue-specific regulation of gene expression during development (Xu et al., 2012; Colquitt et al., 2013; Ge et al., 2014; Madzo et al., 2014; Li et al., 2015). The balance between DNA methylation and demethylation during development is essential for cell differentiation and the generation of normal tissues. Among these, the role of DNA methylation and demethylation in the development of the retina, especially photoreceptors, has been widely reported. In photoreceptors, cone and rod-specific genes exhibit cell-specific DNA methylation patterns (Rao et al., 2011; Chao and Skowronska-Krawczyk, 2020), this suggests that DNA methylation is involved and maintained in the differentiation of retinal cell types. It has also been shown that DNA methylation is increased in dead photoreceptors, suggesting that DNA hypermethylation is a common denominator of photoreceptor degradation pathways (Corso-Diaz et al., 2020). DNA methylation is also involved in the development of diseases such as retinitis pigmentosa (Singh et al., 2018).

In eukaryotic cells, the N- and C-terminal tails of histones can undergo post-translational modifications, such as acetylation, phosphorylation, methylation, SUMO-ization, and ubiquitination (Strahl and Allis, 2000). These post-transcriptional modifications can alter the electronic charge and structure of these DNA-bound histone tails, thereby altering the chromatin state and subsequent gene expression (Kouzarides, 2007). Some histone modifications, such as acetylation and phosphorylation, are reversible and dynamic, and are often associated with the inducible regulation of individual genes. In contrast, histone methylation is more stable and participates in transcription through heritable changes in chromatin conformation. Among them, we mainly discuss histone methylation catalyzed by histone methyltransferase (HMT), which uses S-adenosylmethionine (SAM) as a substrate to transfer methyl groups to lysine residues of histones (Murray, 1964). Histone methylation usually occurs at lysine (K) residues of histones H3 and H4 (Greer and Shi, 2012), which can form mono-, dimethyl- and trimethylation (me1, me2, and me3, respectively) and regulate activation or repression of gene expression. Generally, H3K4, H3K36, and H3K79 methylation are considered markers of transcriptional activation, while H3K9, H3K27, and H4K20 methylation are considered to be associated with transcriptional repression (Black et al., 2012) (as shown in Figure 2B). H3K9me2 and H3K9me3 methylation are the main determinants of constitutive heterochromatin, which consists of chromosomal regions that remain condensed throughout the cell cycle and silence gene transcription. Similarly, trimethylation of H4K20 also participates in the formation of heterochromatin. The mechanism of transcriptional repression mediated by H3K27 methylation is not yet clear, but some studies suggest that H3K27 acquires methylation and transcriptional repression activity through interaction with PcG proteins (which are known transcriptional repressors that play a critical role in regulating various developmental and physiological processes in the cell (Wang et al., 2015)). Modifications of histones are dynamically regulated by various enzymes, including histone demethylases (KDMs), which catalyze the removal of methyl groups from histones. LSD1, a member of the flavin adenine dinucleotide-dependent amine oxidase superfamily, and KDM2A, containing the JmjC structural domain (which is a diverse superfamily of proteins containing a conserved barrel-shaped structure that possesses histone demethylase catalytic activity (Accari and Fisher, 2015)), are two known KDMs that can carry out this process (Shi et al., 2004; Tsukada et al., 2006). Dysregulation or mutations in the enzymes responsible for histone methylation, demethylation, and binding to methylated lysines have been linked to numerous diseases (Greer and Shi, 2012), such as inherited retinal disease (IRD) (Berger et al., 2010) and retinitis pigmentosa (RP) (Zheng et al., 2018). Furthermore, DNA methylation has been shown to be negatively correlated with H3K4 methylation and positively correlated with H3K9 methylation (Hashimoto et al., 2010).

The evidence available suggests that epigenetic methylation and demethylation modifications in photoreceptors are a complex mechanism. However, our understanding of how the epigenetic state of photoreceptors changes sequentially during retinal development and how these mechanisms are involved in different steps of photoreceptor development, such as the proliferation of retinal progenitors, specification of photoreceptor cell fate, expression of photoreceptor-specific genes, and terminal differentiation, is currently limited (Song et al., 2022). Given the critical role of epigenetic regulation in photoreceptor differentiation and function, studying the regulation of these cells by methylation and demethylation modifications may provide a valuable research basis for understanding the development of photoreceptors and identifying potential therapeutic targets for retinal diseases. In particular, elucidating the specific epigenetic mechanisms that govern photoreceptor differentiation, maturation, and maintenance could lead to the development of novel interventions that aim to modulate or restore the normal epigenetic state of these cells in diseased states. As our understanding of the complex interplay between genetic and epigenetic factors in photoreceptor development and function continues to evolve, it is increasingly clear that studying the epigenetic regulation of these cells represents a promising avenue for future research. By leveraging the power of cutting-edge genomic and epigenomic technologies, researchers may be able to gain new insights into the underlying molecular mechanisms that drive photoreceptor differentiation and function, ultimately leading to the development of more effective treatments for a wide range of retinal disorders.

## 2 Characterization of methylation in photoreceptors

### 2.1 Characterization of DNA methylation in photoreceptors

Epigenetic modifications such as DNA methylation play a crucial role in the regulation of gene expression, with methylation typically repressing transcription and demethylation activating transcription (Moore et al., 2013). Within the retina, specific subtypes of cells exhibit distinct DNA methylation patterns during various stages of development (Rao et al., 2011; Chao and Skowronska-Krawczyk, 2020). These patterns likely contribute to the precise fate determination and differentiation of retinal precursor cells.

The process of mouse retinal development involves various stages, each exhibiting distinct patterns of DNA methylation and hydroxymethylation (Singh et al., 2018). At embryonic day 16 (E16), 5-methylcytosine (5 mC) staining was stronger in the chromocenters of the cell nucleus while weaker in the rest of the nucleus, and 5-hydroxymethylcytosine (5 hmC) staining was strongly present in the whole nucleus except for the chromocenters. By postnatal day 0 (P0), 5 mC staining had weakened in the chromocenters and strengthened throughout the entire nucleus, while 5 hmC staining remained similar to E16. At P15, all retinal layers, including the outer nuclear layer (ONL), inner nuclear layer (INL), and ganglion cell layer (GCL), showed strong staining for both 5 mC and 5 hmC. In ONL optic rod photoreceptors, 5 mC signals were found in both the chromocenters and periphery of the nucleus, while 5 hmC signals were present throughout the nucleus except for the chromocenters. These findings suggest that DNA methylation levels undergo significant changes during middle and late stages of retinal development, and such modifications may play a role in subsequent cellular differentiation and functional maturation.

Rod photoreceptor cells in mammals, including mice and humans, constitute the majority of retinal cells, accounting for 70%–80% (Wright et al., 2010). In a study of photoreceptor cell-specific gene methylation levels in 2-month-old C57BL/6J mice, non-photoreceptor cells isolated from the inner nuclear layer (INL) exhibited higher levels of methylation than photoreceptor cells (mainly rods) in the outer nuclear layer (ONL), particularly around the transcription start site (TSS) region of genes encoding retinal-binding protein 3 (RBP3) and rhodopsin (RHO) (Merbs et al., 2012). These genes showed low levels of methylation when compared to those of brain, kidney, and testis. Similar findings have been reported in other studies, where DNA methylation levels in the ONL region of mature mice, where most cells are light receptors, were relatively low (Mears et al., 2001). Furthermore, genes encoding rod-specific proteins (RBP3 and RHO) and cone-specific proteins (OPN1MW and OPN1SW) were found to be essentially unmethylated in the TSS region. Accordingly, hypomethylation has been recognized as a marker for the activation of marker protein genes and a regulatory mechanism for differentiated cells to express their respective specific proteins. During mouse retinal development, the promoters of rod photoreceptor cell-specific genes *Rho* and *Pde6b*, associated with retinitis pigmentosa, exhibit high levels of DNA methylation at postnatal day 2 (P2) but low levels at P28. In contrast, *Nrl*, a determinant of rod photoreceptor fate, consistently displays persistent low DNA methylation throughout the promoter and gene body regions in P2, P10, and P28 rod photoreceptor cells. Rod-specific phototransduction genes, including *Rho*, *Gnat1* (encodes the *α* subunit of the rod transducin protein (Carrigan et al., 2016)), and *Cnga1* (cyclic nucleotide-gated channel protein subunit A1, which plays an important role in the transduction pathways of rod photoreceptors (Liu et al., 2021)), show low levels of DNA methylation and significant upregulation of gene expression during P6 to P10. Conversely, the cone-specific gene *Pde6c*, associated with color blindness and visual cone dystrophy (Jiménez-Siles et al., 2022), consistently exhibits high levels of DNA methylation on gene bodies in P2, P10, and P28 visual cone cells, while the gene coding for the visual cone cell marker protein OPN1MW displays relatively high levels of DNA methylation (approximately 60%) at P28 (Kim et al., 2016).

DNA methylation is dependent on DNA methyltransferases (DNMTs), and the level of DNMTs can reflect the level of DNA methylation in cells to a certain extent. DNMTs show cell specificity, and in the mouse retina, DNMT1 expression is highest during development. The mRNA level is highest at embryonic day 11.5 and gradually decreases with age (Nasonkin et al., 2011). In photoreceptors, lower levels of DNMT1, DNMT3A, and DNMT3B are observed at later stages of retinal development (Nasonkin et al., 2011). The overall low level of DNMTs in the middle and late stages of development suggests that the focal period of DNA methylation regulation of photoreceptor cells lies in pre- or mid-retinal development.

### 2.2 Characterization of histone methylation in photoreceptors

The retina exhibits temporal and spatial-specific expression of histone methylation modifications, which regulate physiological functions in a bidirectional manner. The dynamics of histone methylation during development are complex, and few studies have investigated its levels in photoreceptors. Lysine-specific demethylase 1 (LSD1), a histone demethylase, reflects the level of histone methylation to some extent. LSD1 is highly expressed in late-stage retinal progenitor cells, and inhibition of LSD1 blocks differentiation of mature retinal cells into rods (Popova et al., 2016). In mice, LSD1 is highly expressed from P2 to P14, with differential expression observed in different cell subtypes at P21. By P36, when the retina matures, LSD1 expression differs significantly between layers and between different cells within layers (Ferdous et al., 2019). H3K4me1 and H3K4me2, histone methylation modifications at the H3K4 site, have high levels of expression in P2 and P7, but decrease significantly as the retina fully matures, remaining consistent throughout development in all cells expressed in the retina throughout the mouse’s lifetime. At P36, the outer nuclear layer (ONL) has lower LSD1 levels compared to the ganglion cell layer (GCL) and inner nuclear layer (INL), and LSD1 is mainly expressed in 3% of total ONL cells in cones and low in rods (Ferdous et al., 2019). LSD1 levels exhibit cellular variability in the mouse retina ONL, but the mature human retina has a uniform LSD1 expression pattern in the ONL (Solovei et al., 2009). These findings suggest that histone methylation is a mechanism of retinal development in humans and rodents, but further research is needed.

## 3 Regulation of photoreceptor differentiation by methylation

### 3.1 Regulation of photoreceptor differentiation by DNA methylation

The process of retinal progenitor cell differentiation into photoreceptor cells is a continuous process that can be initiated by the expression of OTX2, an important positive regulator of photoreceptor production (Nishida et al., 2003). Subsequently, the cone-rod homeobox (CRX) is upregulated to direct committed cells to exit the cell cycle and become photoreceptor precursors. CRX plays a crucial role in rod and cone photoreceptor differentiation (Chen et al., 1997; Furukawa et al., 1997; Rhee et al., 2004), while NRL and TRβ2 (encoded by *Thrb*) are two key transcription factors that determine the production of three different types of photoreceptors (rods, S-cones, and M-cones) by postmitotic precursors (Ng et al., 2011). The highly polarized neurons of the outer segments (OSs) in the retina initiate the visual process by using membranes to organize visual pigments and other phototransduction components to capture light quanta. Photoreceptor cells are highly specialized sensory neurons with unique transcriptomes that work together to accomplish specific cellular fates. Key regulatory proteins for photoreceptor development include OTX2, RORβ, BLIMP1 (PRDM1), and CRX (Swaroop et al., 2010). In addition, downstream targets of NRL and CRX and signaling proteins that alter their activity further regulate the expression of photoreceptor-related genes (Hao et al., 2011).

**FIGURE 3:**
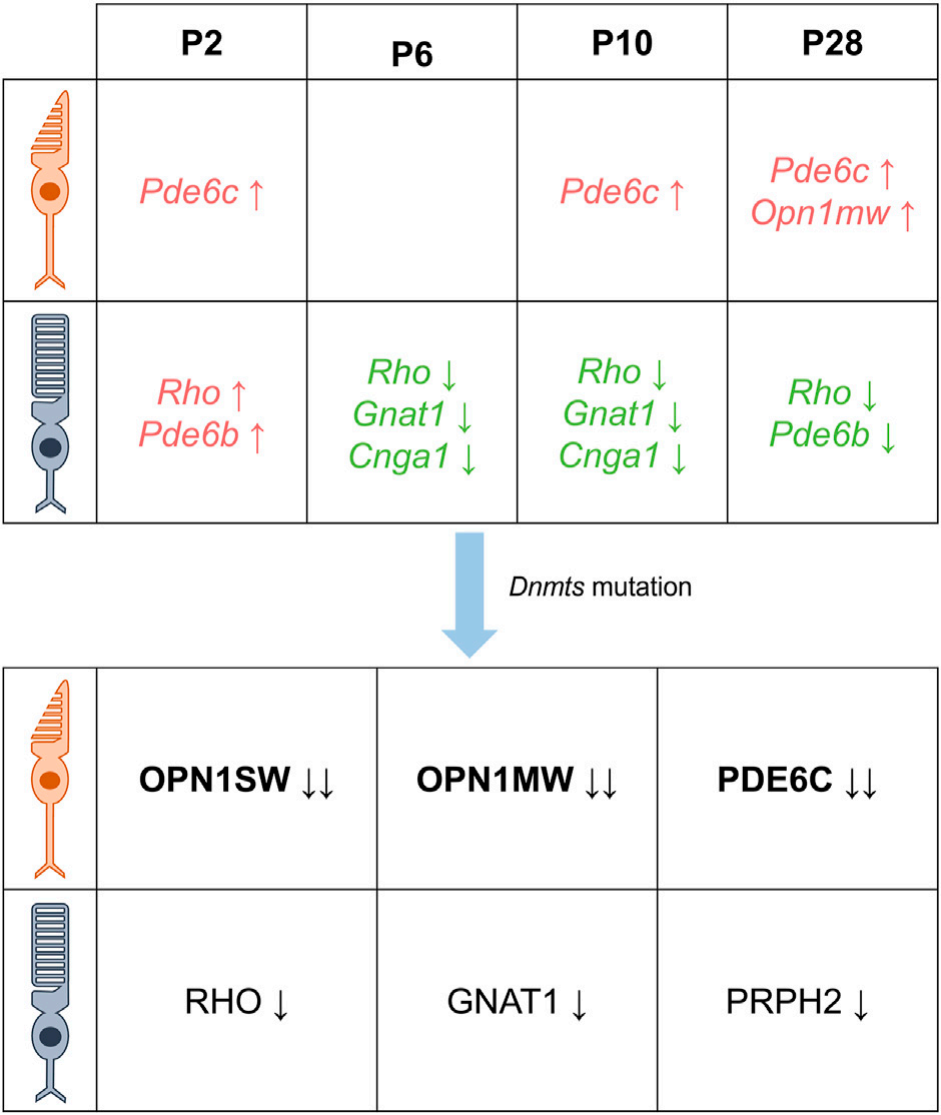
DNA methylation is characterized differently at different times in the photoreceptor. There are differential DNA methylation patterns associated with the expression of cone- and rod-specific genes in the postnatal mouse retina. Specifically, the cone-specific genes *Pde6c* and *Opn1mw* exhibit increased levels of methylation, while the rod-specific genes *Rho, Gnat1, and Cnga1* show lower levels of methylation from 6 days after birth. In addition, mutations in *Dnmt* resulted in decreased protein levels of cone-specific proteins OPN1SW, OPN1MW, PDE6C and rod-specific proteins RHO, GNAT1, and PRPH2, with cones being more severely affected than rods (Green indicates decreased DNA methylation, red indicates increased DNA methylation, black indicates decreased protein levels, the direction of the arrow indicates an increase or decrease, and the number of arrows indicates a change in level.)

Recent studies indicate that DNA methylation plays a crucial role in regulating photoreceptor gene expression during retinal formation (Merbs et al., 2012). Notably, rod and cone photoreceptors derived from common progenitor cells exhibit distinct localization patterns of DNMTs during retina development after mitosis (Nasonkin et al., 2011). Studies involving retina-specific DNMT1 deletion mutations in mice have shown that hypermethylated DNMT1 mutant retinas do not affect the proliferation of retinal progenitor cells but alter the progression of the cell cycle. This alteration is evidenced by an increased proportion of G1 phase cells, highlighting the importance of DNA methylation in photoreceptor differentiation. Interestingly, despite DNA hypomethylation, the normal expression of OTX2 in *Dnmt1*-deficient precursor cells suggests that the fate of photoreceptors remains unaffected. Furthermore, while initiation of *Crx* expression is not affected, its expression occurs in progenitor cells rather than in cells localized near the future outer nuclear layer. Several studies have highlighted the important role of DNA methylation in photoreceptor differentiation. For instance, there is evidence to suggest that DNA methylation defects can interfere with the differentiation of photoreceptor precursors, as evidenced by the co-expression of CRX and progenitor markers, and the mislocalization of CRX^+^ and RHO^+^ cells (Rhee et al., 2012). Further support for this idea comes from bisulfite sequencing analysis of photoreceptor-specific genes, which showed significant hypomethylation of CpG sites in the promoters of *Rho* and *M-opsin* genes at P3 in *Dnmt1* ^fl/fl^ mice (specific knockout of Dnmt1) (Nasonkin et al., 2013). In another study involving Rx-Cre-mediated conditional DNMT1 knockout mice, though no lamination or cell fate defects were observed, a complete lack of photoreceptor outer segments was noted, suggesting an essential role for DNMT1-dependent DNA methylation in photoreceptor differentiation (Rhee et al., 2012). Moreover, studies in *Dnmt* triple mutant mice (knockout of *Dnmt1*, *3a* and *3b*) have shown that these mice exhibit a severe phenotype at P15.5, characterized by alterations in the structure of the photoreceptor layer and the absence of photoreceptor outer segments (Singh et al., 2017). Several studies have investigated the effects of *Dnmt* mutations on opsin expression in rod and cone photoreceptor cells. These studies consistently reported reduced or absent expression of rod-specific opsins (RHO, GNAT1, and PRPH2) and cone-specific opsins (OPN1SW, OPN1MW, and PDE6C) in various *Dnmt* mutants (Rhee et al., 2012; Nasonkin et al., 2013; Singh et al., 2017). In trimutant retinas, OPN1SW was completely absent, while OPN1MW and PDE6C were significantly reduced; in contrast, RHO and PRPH2 levels were not significantly reduced (Singh et al., 2017). Similarly, immunostaining for rod-specific markers (RHO, PRPH2) in DNMT1-specific knockout retinas showed near-normal expression in the inner segment, while PNA staining revealed significant under-expression of cones in the mutant retina, reduced M-cone opsin numbers, and complete absence of S-cone opsin immunostaining (Nasonkin et al., 2013). Overall, these findings suggest that *Dnmt* mutations have a greater effect on cone photoreceptor cells, with S cone cells being particularly sensitive (as shown in Figure 3). However, the role of epigenetics in regulating opsin expression during the development of rod and cone photoreceptors remains unclear (Jones and Baylin, 2002; Esteller, 2007; Otteson, 2011; Peng and Chen, 2011; Rao et al., 2011).

DNA methylation is an essential epigenetic mechanism that requires erasers to remove methyl groups from DNA. Among these erasers, TETs play a crucial role in the demethylation process by adding hydroxyl groups to the methyl group of 5 mC to form 5hmC. In mammals, there are two different mechanisms to convert 5 hmC back to cytosine. Recent studies have highlighted the importance of TET3 in promoting the expression of key developmental genes during eye development in *Xenopus* frogs (Xu et al., 2012). Additionally, the DNA demethylation pathways have been proposed to play a significant role in mouse photoreceptor differentiation (Perera et al., 2015). It has been observed that various *Dnmt* knockout mice exhibit reduced expression of phototransduction genes and lack of photoreceptor *in vitro* segments (Rhee et al., 2012; Nasonkin et al., 2013; Singh et al., 2017). The knockout of the TET protein family results in a similar phenotype, as observed when many photoreceptors and RGC precursors in the zebrafish TET2/TET3 double knockout fail to differentiate into mature neurons. The few photoreceptors that can still differentiate fail to form outer segments (Seritrakul and Gross, 2017). Similarly, knockdown of TET protein family in the mouse retina results in defective ganglion development in the cones and deletion of rods, suggesting that TET protein family may act after the emergence of cone cells and before the emergence of rods. Inactivation of opposing DNA methylation and DNA demethylation pathways leads to the same pathological phenotype during retinal development, which requires further investigation. However, it is possible that DNA methylation and DNA demethylation may be involved in differentiating photoreceptors at different stages, as demonstrated by research showing that promoters of rod, cone, and rod photoreceptor genes were highly methylated in DNA isolated from human and mouse fetal retinas (which mainly contain RPCs) and postnatal mouse RPCs. During the differentiation of RPCs into photoreceptors, the methylation of these promoters was significantly reduced, and the expression of these genes increased (Dvoriantchikova et al., 2019). This suggests that the DNA demethylation pathway is also required for the development of the retinal photoreceptor phenotype. In contrast, the role of DNA methylation and DNA demethylation pathways in the differentiation of RPCs to non-photoreceptor retinal phenotypes may be less critical (Dvoriantchikova et al., 2019). Another study confirmed these findings, indicating that gene promoters essential for rod and cone photoreceptor fate specification and maturation, such as *Crx*, *Nrl*, *Nr2e3*, *Pde6a*, *Pde6g*, *Pde6c*, *Pde6h*, *Gnat1*, *Rho*, *Ush2a*, *Prph2*, *etc.*, are hypermethylated in RPCs and demethylated during the transition from RPCs to photoreceptors (Dvoriantchikova et al., 2022). Additionally, the Wnt signaling pathway can inhibit cellular differentiation in the retina (Kubo et al., 2005). Interestingly, it has been observed that applying Wnt pathway inhibitors after *tet2/tet3* mutant partially repairs the damage to photoreceptors caused by TET deletion (Seritrakul and Gross, 2017), suggesting that epigenetically influenced cellular signaling pathways may also play a role in its regulatory effects.

### 3.2 Regulation of photoreceptor differentiation by histone methylation

Histone post-translational modifications, particularly acetylation and methylation, have been extensively studied as epigenetic marks in the retina. Similar to DNA methylation, genome-wide changes in histone marks during retinal development reveal their crucial role in regulating gene expression. In this context, we focus on the role of histone methylation/demethylation in photoreceptor differentiation. The normal differentiation of photoreceptors requires the downregulation of genes of retinal progenitors and the upregulation of cell type-specific genes, which is associated with genome-wide reductions and increases in the active epigenetic marker H3K4me2 at the transcriptional start site of these genes (Popova et al., 2012). Strong H3K4me2 signaling has been observed at the loci of rod-specific genes (Popova et al., 2012). Additionally, photoreceptor-specific loci were labeled with H3K4me3, but not in non-photoreceptor cells. Moreover, at 2 days after birth, the levels of H3K4me3 in retinal progenitors were similar to those in photoreceptor cells, indicating that photoreceptor-specific H3K4me3 signaling was present before apparent differentiation into photoreceptors (Ueno et al., 2016). Key transcription factors for photoreceptor development, including NRL, CRX, and OTX2, are also involved in H3K4 methylation during retinal development (Kizilyaprak et al., 2010; Ueno et al., 2016; Iwagawa and Watanabe, 2019). In a retinal explant model, depletion of the histone methyltransferase SETD1A produced a hypomethylation pattern of H3K4, resulting in an increase in apoptotic cells and a significant decrease in proliferating cells, as well as a decrease in the number of rod photoreceptor cells during late retinal development (Deng et al., 2021). These findings indicate that the terminal differentiation of retinal cells involves the dynamic regulation of histone methylation, especially the methylation of H3K4.

Lysine-specific demethylase 1 (LSD1), also known as KDM1A (Shi et al., 2004), is a well-studied histone-modifying enzyme that regulates gene expression through the demethylation of H3K4me1/2 and H3K9me1/2. LSD1 does not alter the trimethylation of H3K4 and H3K9 (Shi et al., 2004; Hou and Yu, 2010). High levels of LSD1 are required to maintain the undifferentiated state of human embryonic stem cells (Adamo et al., 2011). In the retina, functional studies using pharmacological inhibitors have identified the role of LSD1 in retinal differentiation (Lee et al., 2006; Schmidt and McCafferty, 2007; Sun et al., 2010; Shi et al., 2013). Expression of LSD1 peaks during the transition from late progenitor cells to rod-shaped photoreceptors in mouse retinas, and inhibition of LSD1 blocks rod-shaped photoreceptor differentiation (Popova et al., 2016). This suggests that LSD1-mediated H3K4me2 demethylation is required for the transition of late progenitor cells to differentiated rod photoreceptors. Interestingly, transcription factors CRX and NRL are expressed normally during this process, and H3K4me2 marks accumulate normally on promoters and genosomes. It is speculated that LSD1 acts synergistically with a series of nuclear receptors to modify chromatin structure in differentiated postmitotic retinal cells and suppress ectopic patterns of gene expression.

G9a (KMT1C) is another histone methyltransferase that acts on histone H3 and methylated H3K9. G9a has been reported to be highly expressed in the developing mouse retina (Katoh et al., 2012). Mice with retinal precursor cell condition knockout of G9a (G9a Dkk3 CKO) exhibited severe morphological defects with loss of photoreceptor cells and sustained cell proliferation (Katoh et al., 2012). However, deletion of G9a did not show a distinct phenotype in photoreceptors using *Crx* promoters, suggesting that G9a appears to play a role in retinal precursor cells (Katoh et al., 2012).

During retinal development, the transcription of specific genes is commonly associated with the corresponding deposition of H3K4me2/3 (associated with activation) and H3K27me3 (associated with repression) (Skowronska-Krawczyk et al., 2004; Usui et al., 2013; Sifuentes et al., 2016; Ueno et al., 2017). Upregulation of Rho and rod cell-related genes was observed in the outer nuclear layer (ONL) of retinas with specific ablation of EZH2, which methylates H3K27. Conversely, overexpression of Rho resulted in photoreceptor degeneration (Olsson et al., 1992; Sung et al., 1994), suggesting that H3K27me3 protects and maintains rod photoreceptor differentiation or maintenance by blocking unwanted or toxic gene expression (Ueno et al., 2016).

## 4 Regulation of photoreceptor death and degeneration by methylation

### 4.1 Regulation of photoreceptor death and degeneration by DNA methylation

Photoreceptor loss or dysfunction can result from genetic mutations, transcriptional disorders, and microenvironmental changes. While programmed cell death during retinal development and degeneration has been extensively studied, little is known about how DNA methylation regulates retinal photoreceptor death. Studies have demonstrated the importance of DNA methylation for the normal differentiation of photoreceptors and have also found that failure of neuronal differentiation can lead to rapid and massive cell death (Corso-Diaz et al., 2018). Recent evidence suggests that DNMT1 mutations can cause progressive neurodegeneration and late-onset neurological disease in humans (Klein et al., 2011; Winkelmann et al., 2012). Decreased DNA methylation has also been observed in age-related macular degeneration (Hunter et al., 2012). *Dnmt1* ^fl/fl^ mice displayed a significant increase in caspase3 signaling throughout the retina, with most retinal cells being lost and the retinal thickness significantly reduced, particularly in the outer nuclear layer. Additionally, an increase in the number of fixation nuclei and reduced Rho expression were observed in DNMT1-deficient cells, indicating that DNMT1 function is essential for the survival of retinal photoreceptors (Rhee et al., 2012).

Retinitis pigmentosa (RP) is a group of inherited degenerative retinal diseases that result in the progressive loss of photoreceptors. RP is the leading cause of severe vision loss and blindness in young people in developed countries (Hartong et al., 2006). In RP, photoreceptors undergo a process of directed cell death characterized by the death of rod photoreceptors followed by secondary loss of cone photoreceptors, ultimately leading to vision loss. Retinitis pigmentosa (RP) is thought to be caused by genetic mutations in a variety of genes, many of which regulate the specification, maturation, and function of rod and cone photoreceptor cells during retinal development (e.g., *Crx*, *Nrl*, *Nr2e3*, *Pde6a*, *Prph2*, *Ush2a*, *Rho*, *etc.*) (Swaroop et al., 2010; Altschwager et al., 2017; Tsang and Sharma, 2018; Gill et al., 2019). While many genes have been identified in association with these diseases, many cases remain unassociated with any genes (Zeitz et al., 2015; Altschwager et al., 2017; Tsang and Sharma, 2018; Gill et al., 2019), suggesting that different mechanisms may be involved in the pathogenesis of RP and related disorders. The high genetic heterogeneity of RP severely limits therapeutic development, necessitating a better understanding of the mechanisms involved. Animal models that reproduce photoreceptor cell loss are useful for exploring the pathophysiology of retinal degeneration. The rd1 mouse model is a good example of such a model for RP. These mice undergo apoptotic-like photoreceptor loss starting at postnatal day 10 (P10) and peaking at P15, resulting in outer nuclear layer thinning. By P21, almost all rod cells have disappeared, leaving only one or two rows of cone cells (LaVail and Sidman, 1974; Portera-Cailliau et al., 1994). Recent studies focusing on cell loss in rd1 mice have shown that the mechanism of cell death in this model is caspase-independent. As a result, current research has shifted from apoptotic processes to non-apoptotic pathways (Chang et al., 1993). Previous microarray studies have demonstrated significant changes in gene expression in the rd1 RP mouse model compared to wild-type animals (Azadi et al., 2006). Epigenetic events are commonly implicated in altered gene expression in various biological processes (Zhou et al., 2011; Corso-Diaz et al., 2018), including those related to the retina. This is because epigenetic mechanisms play a crucial role in the modulation of gene expression, and epigenetic modifications have been shown to regulate gene transcription by modulating the physical state of chromatin, such as its relaxation or condensation. Specifically, epigenetic changes can affect the accessibility of DNA to transcription factors and other regulatory molecules, ultimately influencing the expression of genes involved in different stages of retinal development and function. Therefore, understanding the epigenetic basis of retinal biology can provide valuable insights into the underlying molecular mechanisms of normal and pathological retinal processes.

The role of DNA methylation in retinal neuronal cell death during photoreceptor degeneration in rd1 mice was investigated using immunohistochemical staining for 5-methylcytosine (5 mC) and 5-hydroxymethylcytosine (5 hmC). Our results revealed that an increase in cell-specific 5 mC and 5 hmC immunostaining was associated with the death of retinal neurons during degeneration, highlighting the dynamic regulation of DNA methylation that occurs in parallel with retinal neuronal cell death. Although the genetic targets of this dynamic regulation remain unknown, our findings suggest that the epigenetic regulation of gene expression may play a role in retinal degeneration. These previously unrecognized epigenetic mechanisms could contribute to the onset and/or progression of cell death in the retina (Wahlin et al., 2013).

Recent studies have suggested that DNA methylation plays a role in both retinal degeneration and development (Wahlin et al., 2013). However, it remains unclear how DNA methylation patterns vary across different genes in healthy *versus* diseased tissues, and whether modulating DNA methylation can benefit photoreceptor survival during degeneration. To address these questions, we compared retinal DNA methylation levels between rd1 mouse models of retinitis pigmentosa and healthy homozygous WT mice, correlated DNA methylation changes with gene expression datasets, and further analyzed DNA methylation in three other RP animal models. Our findings revealed that DNA hypermethylation was a common feature of dead photoreceptors in all four RP models, suggesting that DNA methylation dysregulation is associated with photoreceptor degeneration. Additionally, we assessed the effects of DNA methylation inhibitors on rd1 photoreceptor survival *in vitro*. Pharmacological inhibition of DNMTs significantly reduced rd1 photoreceptor cell death in short-term experiments, although no increase in cell survival was observed in long-term experiments. These results suggest that the relationship between DNA methylation and retinal degeneration is complex (Farinelli et al., 2014).

Hypermethylation of DNA can occur in conjunction with other epigenetic events, such as hypoacetylation, which suggests an increase in HDAC activity. Indeed, DNA methylation and HDAC events are known to be correlated (Fuks et al., 2000; Rountree et al., 2000; Aapola et al., 2002), potentially reinforcing each other to block transcription (Kim et al., 2003). In the context of retinal degeneration, it has been proposed that a coordinated epigenetic program may be triggered in dying photoreceptor cells, including DNA remethylation as a means of shutting down transcription and protein biosynthesis to reduce energy consumption during cell death.

Recent research has highlighted the role of genetic mutations in retinitis pigmentosa and related photoreceptor dystrophies (RPRPD), which can result in photoreceptor death and subsequent vision loss. However, emerging evidence also suggests that epigenetic modifications may contribute to the pathogenesis of these disorders. Specifically, unsuccessful demethylation of regulatory sequences (e.g., promoters, enhancers) during the transition from retinal progenitor cells to photoreceptors may reduce or eliminate the activity of genes required for photoreceptor development, maturation, and function, potentially leading to RPRPD even in the absence of genetic mutations (Dvoriantchikova et al., 2022). While further study is needed to confirm the extent of epigenetic contributions to RP and RPRPD, these findings suggest a potential new avenue for therapeutic intervention.

### 4.2 Regulation of photoreceptor cell death and degeneration by histone methylation

Recent research has demonstrated that modulation of histone methylation/demethylation can have a protective effect against photoreceptor cell death and degeneration in pathological conditions (Zheng et al., 2018; Popova et al., 2021; Miller et al., 2022). In a cpfl1 mouse model with a cone-specific *Pde6c* gene mutation, retinal cone loss was observed at P14, followed by peak cell death at P24, which was associated with reduced levels of H3K27me3 in retinal cones (Trifunović et al., 2010). Treatment of this model with the H3K27 histone demethylase (HDM) inhibitor GSK-J4, which targets KDM6A, KDM6B, and KDM5 subfamilies (with KDM5 responsible for demethylation of H3K4me1/2/3 (Heinemann et al., 2014)), resulted in a significant increase in optic cone photoreceptor numbers, suggesting a potential role for H3K27me3 deletion in cone degeneration (Miller et al., 2022). By contrast, in the rd1 mouse model of RP, deletion mutations in the rod-specific *Pde6β* gene led to increased H3K27me3 levels in postnatal retinas, while treatment with the HMT inhibitor DZNep delayed rod death by downregulating NRL and its downstream target NR2E3 to protect rods (Zheng et al., 2018). In another study using the rd1 mouse model, inhibition of LSD1 also prevented rod degeneration (Popova et al., 2021). However, the effects of histone methylation appear to be cell type-specific and context-dependent, with the same modification potentially playing opposing roles in different cell subtypes. Therefore, further studies on the precise regulation of histone methylation are essential for gaining insight into the pathogenesis of various retinal diseases.

## 5 Discussion

In this review, we examine the contribution of two key epigenetic mechanisms—DNA methylation and histone methylation—to the differentiation of retinal photoreceptors and the death of photoreceptors in diseases such as retinitis pigmentosa. Previous research has shown that manual interventions for epigenetics often result in failure of cell differentiation and even cell death, as well as animal death before and after birth (Rhee et al., 2012; Nasonkin et al., 2013; Singh et al., 2017), underscoring the crucial role of epigenetics in these processes. Most previous studies investigating the localization and expression levels of DNA methylation and histone methylation during normal retinal development have employed staining methods (Singh et al., 2018; Ferdous et al., 2019). However, these methods have limitations in visualizing and continuously demonstrating epigenetic features in the retina across different developmental stages, hindering our understanding of the complex mechanisms involved. Animal models of relevant photoreceptor diseases have proven effective in mimicking the associated disease phenotypes. Nonetheless, as the specific pathogenesis of these diseases remains poorly understood, it is unclear whether the pathogenesis of gene-deficient mice used in these models accurately reflects that of the simulated disease. It is also uncertain whether the interventions applied to mitigate the disease model can be correctly extrapolated to human cases, despite some mouse mutations being in identical genes as in human RP and displaying similar pathophysiology.

In recent years, the advent of big data and bioinformatics technologies has paved the way for genome-wide analysis in predicting and diagnosing retinal development and diseases. However, to fully characterize the mechanisms of cell fate commitment during retinal development, the impact of genetic variation on retinal photoreceptor disease-related pathways, and environmental influences on phenotype, it is crucial to identify epigenetic contributions to retinal photoreceptor gene regulation. Dynamic methylation plays a critical role in both retinal photoreceptor differentiation and photoreceptor degeneration and death in retinal diseases. As such, investigating the functions of DNA methylation/demethylation and histone methylation/demethylation during retinal development may lead to new treatment strategies for retinal diseases. While considerable progress has been made in recent years in understanding retina-related epigenetic mechanisms, our knowledge about whether and how these epigenetic regulatory mechanisms contribute to retinal photoreceptor differentiation and death in disease remains limited. Although significant advances have been made in studying the epigenetic patterns during development, our understanding of how chromatin structure and the epigenome affect transcriptional mechanisms in disease processes is still lacking. Most previous studies on DNA methylation in the retina, photoreceptor, and blood have not employed genome-wide methylation analysis or examined genome-wide methylation changes with nucleotide resolution during specific diseases or aging, which makes it challenging to elucidate the underlying mechanisms involved.

Currently, most research findings on epigenetic regulation in the retina are limited to *in vitro* and *in vivo* animal studies. However, given the substantial differences in epigenetic regulation between human and animal retinas (Solovei et al., 2009), there is a need for future human studies to better understand the role of epigenetics in retinal development and disease. To gain a more comprehensive understanding of the underlying mechanisms involved in epigenetic regulation, it is essential to investigate the interaction of DNA methylation with other factors in greater depth. While we know that epigenetic regulation plays a critical role in retinal development and eye diseases, much remains to be discovered through ongoing research.
